# Developing indicators of age-friendly neighbourhood environments for urban and rural communities across 20 low-, middle-, and high-income countries

**DOI:** 10.1186/s12889-021-12438-5

**Published:** 2022-01-13

**Authors:** Emily J. Rugel, Clara K. Chow, Daniel J. Corsi, Perry Hystad, Sumathy Rangarajan, Salim Yusuf, Scott A. Lear

**Affiliations:** 1 Faculty of Health Sciences, Simon Fraser University, Burnaby, BC Canada; 2 Westmead Applied Research Centre (WARC), Faculty of Medicine and Health, The University of Sydney, Sydney, NSW Australia; 3 School of Epidemiology and Public Health, University of Ottawa, Ottawa, ON Canada; 4 College of Public Health and Human Sciences, Oregon State University, Corvallis Oregon, USA; 5 Population Health Research Institute, Hamilton, ON Canada; 6 Department of Medicine, McMaster University and Hamilton Health Sciences, Hamilton, ON Canada; 7 Community Health Research Team, St. Paul’s Hospital, Room 180-57A, 1081 Burrard Street, Vancouver, BC V6Z 1Y6 Canada

**Keywords:** Green space; Walkability; Transportation access; Community health services; Social participation; Healthy ageing; Methodological study design; International collaboration

## Abstract

**Background:**

By 2050, the global population of adults 60 + will reach 2.1 billion, surging fastest in low- and middle-income countries (LMIC). In response, the World Health Organization (WHO) has developed indicators of age-friendly urban environments, but these criteria have been challenging to apply in rural areas and LMIC. This study fills this gap by adapting the WHO indicators to such settings and assessing variation in their availability by community-level urbanness and country-level income.

**Methods:**

We used data from the Prospective Urban and Rural Epidemiology (PURE) study’s environmental-assessment tools, which integrated systematic social observation and ecometrics to reliably capture community-level environmental features associated with cardiovascular-disease risk factors. The results of a scoping review guided selection of 18 individual indicators across six distinct domains, with data available for 496 communities in 20 countries, including 382 communities (77%) in LMIC. Finally, we used both factor analysis of mixed data (FAMD) and multitrait-multimethod (MTMM) approaches to describe relationships between indicators and domains, as well as detailing the extent to which these relationships held true within groups defined by urbanness and income.

**Results:**

Together, the results of the FAMD and MTMM approaches indicated substantial variation in the relationship of individual indicators to each other and to broader domains, arguing against the development of an overall score and extending prior evidence demonstrating the need to adapt the WHO framework to the local context. Communities in high-income countries generally ranked higher across the set of indicators, but regular connections to neighbouring towns via bus (95%) and train access (76%) were most common in low-income countries. The greatest amount of variation by urbanness was seen in the number of streetscape-greenery elements (33 such elements in rural areas vs. 55 in urban), presence of traffic lights (18% vs. 67%), and home-internet availability (25% vs. 54%).

**Conclusions:**

This study indicates the extent to which environmental supports for healthy ageing may be less readily available to older adults residing in rural areas and LMIC and augments calls to tailor WHO’s existing indicators to a broader range of communities in order to achieve a critical aspect of distributional equity in an ageing world.

**Supplementary Information:**

The online version contains supplementary material available at 10.1186/s12889-021-12438-5.

## Background

The world’s population is becoming increasingly concentrated in urban environments and composed of older individuals. Both trends are accelerating rapidly: the global urban population is projected to increase by nearly 60% between 2018 and 2050 to a total of 6.7 billion inhabitants [1], while the number of individuals aged 60 and over is expected to more than double from 1 to 2.1 billion over this timeframe [2]. Much of this doubling in the older-adult population is due to a significant demographic shift occurring in low- and middle-income countries (LMIC), where the proportion of adults aged over 65 is growing three-and-a-half times faster than it is in high-income countries [3].

In response to these dramatic changes, the World Health Organization (WHO) has declared 2020–2030 to be the “Decade of Healthy Ageing" and has focused on intersectoral collaboration to ensure older adults maintain optimal functioning across their lifespans, regardless of gender, socioeconomic status, or country of residence [4]. The WHO’s definition of healthy ageing acknowledges individuals may have one or more chronic health conditions at this stage of life, but these conditions should only minimally restrict core activities, including cognition, mobility, and social participation [5]. This definition also highlights the centrality of the relationship between individual capacities and influencing environments, at every level from the home to broad social policies and programs [5].

Although much of the discourse around healthy ageing focuses on maintaining individual capacities by reducing risk behaviours and supporting chronic-disease management, the built [6, 7], natural [8, 9], and social environments [10, 11] all play significant roles. Seeking to broaden the focus of healthy ageing from clinical care to upstream interventions, numerous organizations have created indicators of “age-friendly” environments, most notably those first described by the WHO in 2007 for specific application to urban environments [12]. To make this broad policy guidance more applicable to urban planning and municipal policy, the WHO subsequently developed a set of core indicators in 2015, reframing the initial model into three principal areas: measures to advance equity, aspects of an accessible physical environment, and features of an inclusive social environment [13].

Unfortunately, these indicators have proven challenging to implement, particularly in countries with varying national-income levels and across the urban–rural gradient. An attempt to apply the core WHO indicators to data from the China Health and Retirement Longitudinal Study (CHARLS) while exploring age-friendliness by degree of urbanness found these indicators to be “heavily urban oriented and industrial centric” [14]; a similar study in Nairobi, Kenya reported they required substantial modification to fully reflect the challenges faced by older adults residing in informal settlements (sometimes referred to as “slums”) [15]. Similarly, the Canadian Age-Friendly Rural and Remote Communities Initiative collected data in ten rural communities and found communities prioritized distinct aspects of their environments in comparison to the factors highlighted in the WHO’s urban-based indicators [16].

Our study seeks to clarify the extent to which the eight WHO domains and 68 associated indicators are applicable across urban and rural areas in low-, middle-, and high-income countries, representing a range of cultural, social, and economic characteristics. The first aim is to develop a robust, novel set of healthy-ageing indicators aligned with the WHO framework by integrating environmental exposure data from a global, longitudinal epidemiological study. The second aim is to describe systematic variation in the availability of these healthy-ageing indicators across a broad and diverse sample of communities.

## Methods

### Community-level environmental assessments

The data for the analysis that forms the basis of this study originate in the Prospective Urban and Rural Epidemiology study (PURE), a longitudinal cohort study examining community, household, and individual behaviours and risk factors for cardiovascular disease (CVD) that began enrolling participants between the ages of 35 and 70 from urban and rural communities starting in 2003 [17]. PURE study communities were purposively selected from low-, middle-, and high-income countries in multiple regions of the world, chosen for their heterogeneity with respect to social, political, and economic contexts. While the precise construction of a PURE “community” differs from country to country, they were broadly defined as “groups of individuals sharing common characteristics and residing in a defined geographic area” and generally aligned spatially with existing administrative boundaries [18]. In urban areas, PURE communities are typically represented by naturally occurring neighborhood areas; in rural areas, communities are represented by small village locations.

The specific indicators integrated here come from a pair of tools designed to reliably describe community-level environmental features specifically associated with CVD risk factors across such a diverse set of communities [19]. The Environmental Profile of a Community’s Health 1 tool (EPOCH 1) relies on systematic social observation (SSO) by trained local research team members who completed a checklist carried out on a one-kilometre walk in the community’s centre that included assessments of tobacco, grocery, and restaurant outlets with the ultimate aim of assessing four distinct environmental domains: tobacco, physical activity, food and alcohol, and social and economic [19]. The other tool, EPOCH 2, was a survey-based instrument that applied an ecometric approach [20] to aggregate responses from PURE study participants into a similar set of environmental scales at the community level [18]. Respondents to the EPOCH 2 survey represented a convenience sample, with 30 participants in each community, equally divided among men and women, from a subset of PURE study members within the community [18].

 During the initial development and validation process, both EPOCH tools were evaluated for their feasibility and validity in a subset of PURE communities (93 for the former and 84 for the latter) across five countries selected to represent the broader range of study sites. The 13 scales in EPOCH 1 were found to have acceptable inter-rater reliability across all 38 included measures, with little variation seen in reliability by country or level of urbanness [19]. EPOCH 2 reported reliabilities of 0.86 to 0.93 for each scale; a multilevel confirmatory factor analysis (CFA) also supported the internal consistency of the scales [18].

### Identification and selection of community-level healthy-ageing indicators

To develop a robust set of healthy-ageing indicators relevant to multiple constructions of healthy ageing across both urban and rural areas, we carried out a scoping review to identify audit tools aligned with the WHO age-friendly cities framework or applied to either rural areas or low- and middle-income countries. The WHO framework was developed via interviews with older adults, caregivers, and service providers from 33 cities across 23 nations and specified eight distinct domains of age-friendly cities, but was explicitly presented as “neither technical guidelines nor design specifications” [12]. Table 1 presents the results of this review, briefly describing each of the seven identified tools that met these criteria, as well as the EPOCH 1 and EPOCH 2 instruments, identifying the setting, detailing the data-collection methodology, and summarizing the principal domains along with individual elements related to the built, natural, and social environments.

**Table 1:** Overview of environmental audit tools for healthy ageing in rural areas and low- and middle-income countries

**Instrument**	**Setting**	**Data Collection**	**Components**
WHO Global Age-Friendly Cities Framework [12]	33 cities located across Argentina, Australia, Brazil, Canada, China, Costa Rica, Germany, Ireland, Italy, India, Jamaica, Japan, Jordan, Kenya, Lebanon, Mexico, Puerto Rico, Pakistan, Russia, Switzerland, Turkey, United Kingdom, United States	Subjective reports from older adults, their caregivers, and service providers collected via focus groups	**Outdoor spaces and buildings**: aesthetics, natural spaces, safety, accessibility, pavement quality, toilets, seating **Transportation:** network connectivity, service frequency, priority seating/parking, sheltered stops/stations **Housing:** affordability, quality, maintenance, location near services and facilities **Social participation:** nearby community centres **Respect and social inclusion** **Civic participation and employment** **Communication and information:** universal written and broadcast media **Community support and health services:** healthcare providers, health promotion services, home care
WHO Global Age-Friendly Cities Core Indicators [13]	15 communities located across Argentina, Australia, China, France, India, Iran, Italy, Kenya, Russia, Spain, United Kingdom, United States	Objective data (SSO, administrative records, governmental statistics, expenditure reports, legal records) ***or*** subjective reports by older adults collected via surveys	**Walkability:** acceptable pedestrian paths ***or*** suitability for walking **Public spaces and buildings:** accessibility by wheelchairs ***or*** accessibility among those with mobility, vision, or hearing limitations **Transportation:** public transit with designated places ***or*** accessibility among those with mobility, vision, or hearing limitations; transit stops within 500-m walking distance ***or*** accessible **Housing:** affordability, based on < 30% of income ***or*** affordable neighbourhoods **Inclusive social environment:** older adult participation in cultural events and facilities ***or*** weekly participation in sociocultural activities **Information:** available information about health concerns and service referrals ***or*** knowing whom to contact about these matters **Social and health services:** formal home- or community-based personal-care services ***or*** having personal-care needs formally met within home or community
Neighbourhood Design Characteristics Checklist [NeDeCC] [21]	England, across a “wide variety of rural–urban environments”	Objective systematic social observation (SSO) conducted by researchers based on an assessment of area within 300 m of older adult participants’ homes	**Housing:** type, form, height, age **Street:** type, shape, pedestrian-traffic segregation, topography, “eyes on the streets”, variety, block size, traffic level, setback **Neighbourhood:** urbanness, predominant block size, street network, intersection density, open space amount, land-use mix, density, “natural surveillance”, “legibility”, traffic, greenery amount
Older People’s External Residential Assessment Tool [OPERAT] [22]	405 postcode areas across Wales, “purposively selected for socio-economic and geographical diversity”	Objective systematic social observation (SSO) conducted by a sole researcher walking through each postcode area; initial set of measures refined via a qualitative thematic analysis of feedback from a 15-member expert advisory group and a set of weights derived from questionnaires distributed to 13 forums of ages 50-plus in Wales	**Natural elements:** public grasses or verges, sounds of nature, private tree density **Incivilities and nuisance:** traffic, industrial, or other loud noises, litter/waste/broken glass, traffic level **Navigation and mobility:** road-sign legibility, street and alley lighting, sidewalk/footpath quality, road quality **Territorial functioning:** building and garden maintenance, parking, industrial nature of main street
WHO Age-Friendly Cities Indicators in informal settlements [15]	Korogocho and Viwandani, two informal settlements in Nairobi, Kenya	Objective data derived from surveys (Nairobi Health and Demographic Surveillance System and Urbanization, Poverty, and Health Dynamics) and from SSO carried out by researchers; subjective reports by older adults drawn from focus groups	**Walkability:** acceptable pedestrian paths **Public spaces and buildings:** new and existing public spaces accessible by wheelchair **Transportation:** public transit with designated places for older people or people with disabilities **Housing:** affordability, based on < 30% of income **Inclusive social environment:** monthly or twice-monthly attendance at group, club, society, union, or organization ***or*** at-least weekly attendance at religious services **Information/Social and health services:** lack of access to healthcare when needed over past year
China Health and Retirement Longitudinal Study [CHARLS] age-friendly criteria [14]	301 rural villages and 152 urban communities across China	Objective SSO data collected by researchers and subjective reports drawn from face-to-face interviews with local officials	**Outdoor spaces and buildings**: topography, road types/quality/cleanliness, public toilets, pollution, road network/cleanliness, construction types, crowds, accessibility **Transportation:** bus lines, distance to bus stops, distance to train stations **Housing:** residential-lot size, community central heating, sewer system, affordability **Social participation:** presence of public exercise facilities/support organizations/arts organizations/activity centres **Communication and information:** telephones, cell phones, TVs **Community support and health services:** presence and distance to public facilities, community centre presence/services/usage/costs
WHO Study of Global AGEing and Adult Health (WHO-SAGE) built environment index [23]	42 districts across South Africa	Objective healthcare data drawn from the District Health Barometer and subjective reports from South African General Household Survey respondents	**Housing:** housing, water, sanitation, electricity **Community support and health services:** nurse clinical workload in primary healthcare facilities
Environmental Profile of a Community’s Health [EPOCH] 1 [19]	652 urban and rural communities in Argentina, Brazil, Canada, Chile, China, Colombia, India, Iran, Kazakhstan, Malaysia, Pakistan, Palestine, Philippines, Poland, Russia, Saudi Arabia, South Africa, Sweden, Tanzania, Turkey, United Arab Emirates, Zimbabwe	Objective systematic social observation (SSO) conducted by researchers during a one-kilometre walk around each community’s centre	**Outdoor spaces and buildings**: sidewalk completeness and quality**,** presence of street trees and flowerbeds, access to public parks and recreational areas, number of public places for recreation/physical activity, road completeness and quality, street lighting, traffic lights **Transportation:** availability and frequency of bus and train connections to other towns, access to train stations **Housing:** cost of residential land, cost of housing **Civic participation and employment:** access to government buildings **Community support and health services:** access to hospitals, access to public and private medical clinics
Environmental Profile of a Community’s Health [EPOCH] 2 [18]	605 urban and rural communities in Argentina, Brazil, Canada, Chile, China, Colombia, India, Iran, Malaysia, Pakistan, Philippines, Poland, Russia, Saudi Arabia, South Africa, Sweden, Tanzania, Turkey, United Arab Emirates, Zimbabwe	Subjective reports by study participants, aged 35–93	**Social participation:** community social cohesion **Communication and information:** internet access at home, free public internet access

### Analytic procedures

**Fig. 1:**
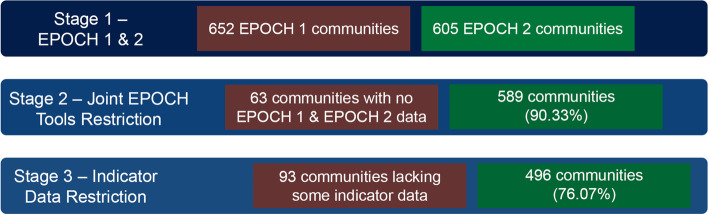
Analytic-sample development by stage

In order to include the broadest set of indicators in alignment with the WHO age-friendly cities framework and earlier work carried out in rural areas and low- and middle-income countries, the lead authors (EJR, CKC, and SAL) examined the results of the limited review to identify an initial set of potentially relevant EPOCH variables (see Supplemental Table 1). Next, data from the EPOCH 1 and 2 tools were merged in R version 4.0.5., with all subsequent quantitative analyses carried out using this software platform [24]. At the time of data extraction in May 2020, EPOCH 1 data were available for 652 communities and EPOCH 2 data were available for 605; a total of 589 communities had at least some data available for both instruments (refer to Fig. 1 for a flowchart representing data losses at each stage of the analysis).

After merging these datasets and pruning variables with a high percentage of missing data (pre-specified as ≥ 20% missing across all study communities), the remaining indicators were summarized via descriptive statistics, using proportions for binary variables and means for both continuous and categorical variables, all of which were based on Likert-scale-like items. These statistics were also calculated for groups of communities defined by community-level urbanness (rural vs. urban) and country-level income category (low-income [LIC], lower-middle income [LMIC], upper-middle-income [UMIC], and high-income [HIC]).

Next, two distinct approaches were carried out to examine the relationship between individual indicators and related domains, both across the sample as a whole and within groups defined by urbanness and income. The first approach applied a data-reduction method known as factor analysis of mixed data (FAMD) using R’s *FactoMineR* package, which combines principal component analysis (PCA) for continuous variables and multiple correspondence analysis (MCA) for categorical variables to allow for the integration of both variable types into a single index [25]. The second approach used a multitrait-multimethod (MTMM) approach [26] via the *mtmm* function in R’s *psy* package [27] to clarify the extent to which our study data conformed with the pre-existing domains identified by the WHO. The combination of these two approaches was selected because they have distinct, but complementary, advantages. MTMM is a robust method of assessing construct validity when comparing a novel set of measures, in this case the EPOCH 1 and EPOCH 2 tools, to a well-described set such as the WHO’s age-friendly framework [28]. Confirmatory factor analysis approaches such as FAMD, on the other hand, are designed to identify latent relationships among variables within a dataset independent of any comparison to a standard, and have been used in the development of healthy-ageing indices that sit outside of the WHO AFC [29]. Finally, by examining and qualitatively integrating the results of both analyses, the three lead authors independently assessed the assignment of individual variables to each of the eight original WHO domains, reaching a final determination via consensus.

## Results

### Community-level healthy-ageing indicator availability

Of the 23 indicators initially identified via consensus from the list of EPOCH 1 and EPOCH 2 tools in alignment with the WHO indicators (see Supplemental Table 1), five were removed due to high levels of missing data: 1) sidewalk quality; 2) daily bus frequency; 3) daily train frequency; 4) cost per unit of residential land; and 5) average housing cost. After removing communities that lacked data for any of the remaining 18 indicator variables, a total of 496 communities contributed data to the analyses presented here, representing three-quarters of all PURE study communities with at least some EPOCH 1 and EPOCH 2 data in May 2020 (see Fig. 1). Communities included in the subsequent analyses were located in the following 20 countries spread across eight regions: Argentina, Brazil, Canada, Chile, China, Colombia, India, Iran, Malaysia, Pakistan, Philippines, Poland, Russia, Saudi Arabia, South Africa, Sweden, Tanzania, Turkey, United Arab Emirates, and Zimbabwe.

### Community-level healthy-ageing indicator values

Table 2 summarizes community-level healthy-ageing indicator values by community-level urbanness and country-level income. Examining measures within the domain of ***outdoor spaces and buildings***, access to natural spaces was relatively high, with 91% of communities having access to parks or other recreational facilities and the number of street trees and flowerbeds averaging a sum of just over 45 on the researchers’ one-kilometre SSO walks. The vast majority of communities had street lighting (91%), but only 45% had traffic lights. Within the domain of ***transportation***, stark differences were observed in transit connections to neighbouring towns via public buses as compared to public trains: 82% of communities offered the former, while only 18% the latter. Looking instead at the presence of a train station within 20 kms of a community’s centre, however, just over half of all communities had access. In terms of the sole measure of ***social participation*** available, community social cohesion was moderate, with an average of 1.9 on a scale of 1 to 4, on which values of “1” correspond to “it is common for people in my neighbourhood to help others” and “4” to helping others “would not occur in my neighbourhood”. In the domain of ***civic participation and employment***, access to government buildings was over 90%. In terms of ***communication and information***, respondents reported having home internet infrequently (an average of 41% of respondents in each community), and free public internet was even rarer, at just 9%. Finally, examining ***community support and health services***, over half of communities contained a hospital (60%), and the presence of a public medical clinic was even more frequent (85%).

**Table 2:** Community-level healthy-ageing indicator values by community-level urbanness and country-level income^a^

	**Country-level Income Category**					**Urbanness**	
**Indicator**	All (496)	LIC (83)	LMIC (168)	UMIC (131)	HIC (114)	Rural (219)	Urban (277)
**Outdoor Spaces and Buildings**							
Sidewalk completeness [1 = no sidewalk; 4 = complete]	2.8	2.2	2.7	2.9	3.3	2.1	3.3
No. of street trees & flowerbeds on 1 km walk	45.2	19.6	48.2	77.7	22.0	32.5	55.2
Access to public parks & recreation areas (%)	91.3	80.7	87.5	97.7	97.4	83.6	97.5
Number of physical-activity facilities on 1 km walk	0.4	0.2	0.3	0.9	0.3	0.5	0.4
Road completeness [1 = none paved; 4 = all paved]	2.8	2.5	2.8	2.7	3.0	2.6	2.9
Road quality [1 = poorly-maintained; 4 = well-maintained]	3.3	3.0	3.1	3.2	3.8	3.5	2.9
Street lighting	90.9	83.1	83.9	97.7	99.1	84.9	95.7
Traffic lights	45.4	24.1	28.0	55.0	75.4	17.8	67.1
**Transportation**							
Availability of buses	81.7	95.2	75.6	89.3	71.9	78.1	84.5
Availability of trains	17.5	22.9	12.5	26.0	11.4	7.8	25.3
Access to train stations	50.8	75.9	30.4	47.3	66.7	27.9	69.0
**Social Participation**							
Community social cohesion [1 = highest; 4 = lowest]	1.9	1.8	1.7	2.1	1.9	1.7	2.0
**Civic Participation and Employment**							
Access to government sites	93.8	96.4	85.1	97.7	100	88.6	97.8
**Communication and Information**							
Availability of home internet	40.9	9.3	27.2	35.9	90.0	24.7	53.8
Availability of free public internet	9.0	2.7	2.4	1.4	33.6	7.0	10.5
**Community Support and Health Services**							
Access to hospitals	60.1	96.4	44.0	61.8	55.3	37.9	77.6
Access to public medical clinics	85.1	86.7	68.5	96.2	95.6	81.7	87.7
Access to private medical clinics	77.8	96.4	64.3	75.6	86.8	57.5	93.9

^a^Numeric and categorical variables are expressed as means; binary variables are expressed as percentages

### Variation by community-level urbanness

There were marked differences between urban and rural communities (as shown in Table 2), highlighted by a few indicators that were only present in a very small proportion of rural communities. These include traffic lights (18%) in ***outdoor spaces and buildings***; train connections (8%) in ***transportation***; and free public internet access in ***communication and information*** (7%). Looking at a different aspect of ***outdoor spaces and buildings***, urban areas had 68% more street trees and flowerbeds on average than rural areas (55 vs. 33); within ***community support and health services***, the percentage of rural communities with access to a public medical clinic was similar to that found across urban areas (82% vs. 88%); and in terms of ***social participation***, participants in rural communities reported a stronger sense of social cohesion (1.7 vs 2.0). Each of these indicators with the greatest amount of variation by urbanness is drawn from a distinct domain, and all six of the included domains are represented.

### Variation by country-level income

As with community-level urbanness, there was a great deal of variation by country-level income, with high-income countries generally ranking higher overall (see Table 2). This finding was not consistent, however. For instance, in the ***transportation*** domain, the percentage of communities with bus connections was greatest in low-income countries, at 95%, versus just 72% in high-income countries; LIC also had the highest rate of access to train stations (76%). In the domain of ***civic participation and employment***, 100% of communities in high-income countries had access to a government site, but such access was generally high regardless of national income level, at 94% overall. Looking at ***communication and information***, just 9% of communities in low-income countries provided home internet access, while 90% of communities in high-income countries did. Free public internet access showed a similar trend by income level, but with much lower rates ranging from 1 to 34%. Finally, in terms of ***community support and health services***, access to hospitals was most common in low-income countries, at 96%, and significantly less frequent in high-income countries, at 55%. This pattern did not hold true for access to medical clinics, for which lower-middle income countries had the lowest rates of both private (64%) and public clinics (69%).

### Alignment between individual variables and broad domains

**Fig. 2:**
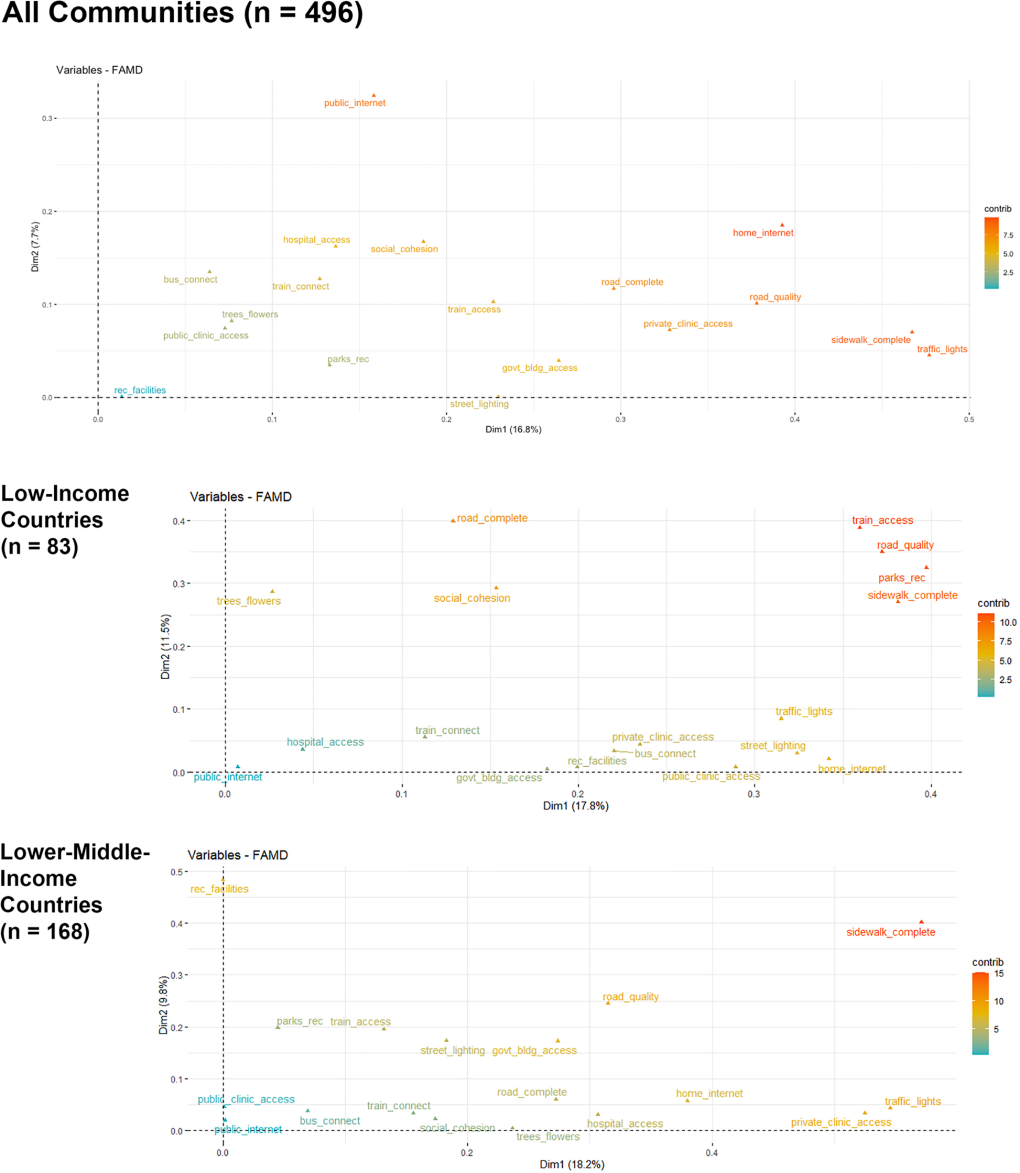

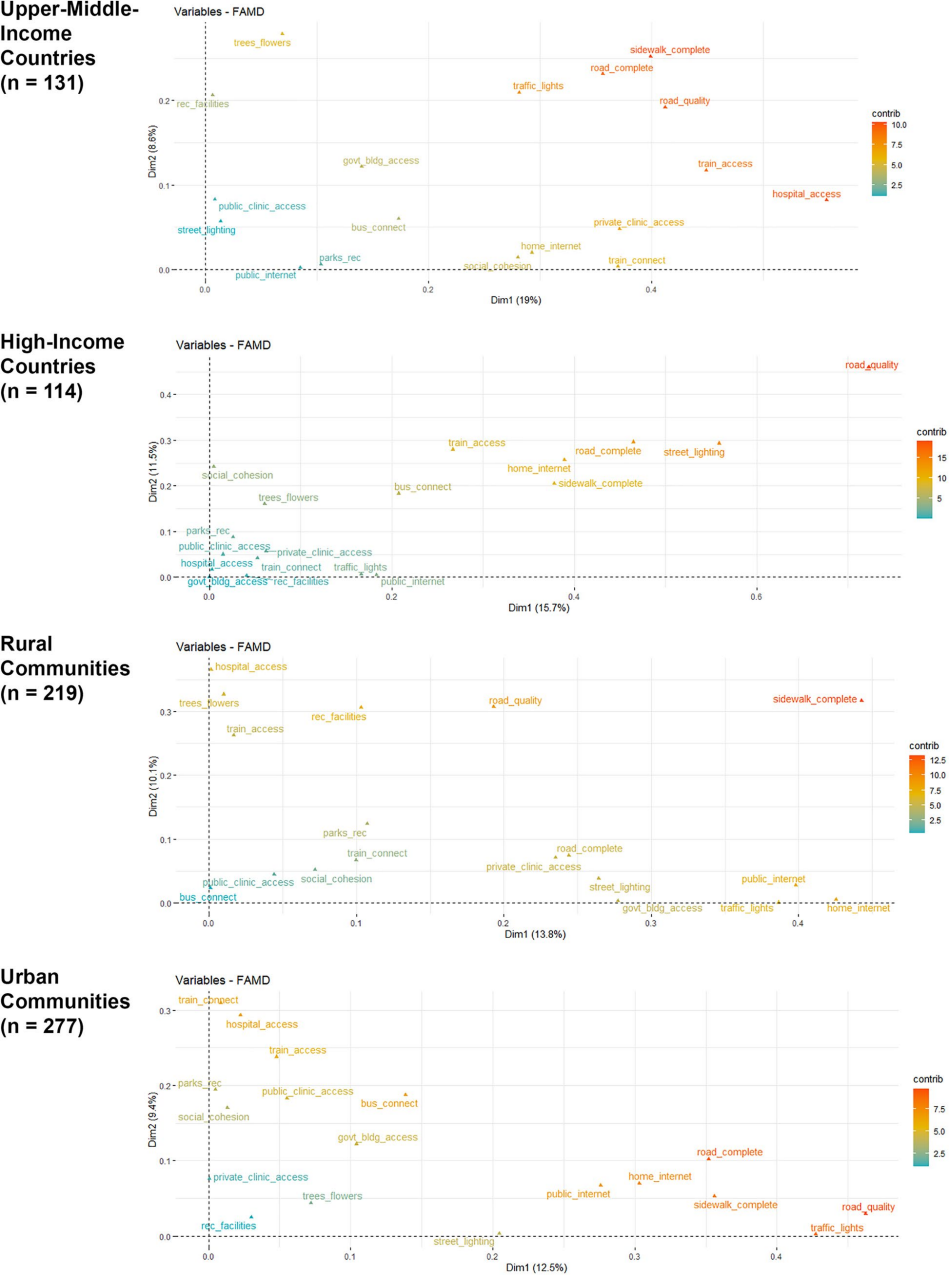
Contributions of individual indicators to Domains 1 and 2 in FAMD

The results of the factor analysis of mixed data approach demonstrate a relatively modest amount of variance explained by the first eight domains (customarily called “dimensions” in FAMD, but referred to as “domains” hereafter), ranging from 57% in the full sample to 69% when integrating data solely from the 114 communities located in high-income countries (see Table 3). In addition, there was substantial overlap in the indicators that contributed the most to each domain regardless of community-level urbanness or country-level income, but the specific indicators with the strongest contributions varied across groups defined by these characteristics (see Fig. 2).

**Table 3:** Cumulative variance explained by FAMD^a^ domains

**Domain**	**Country-level Income Category**					**Urbanness**	
	All (496)	LIC (83)	LMIC (168)	UMIC (131)	HIC (114)	Rural (219)	Urban (277)
Domain 1	16.8	17.8	18.2	19.0	15.7	13.8	12.5
Domain 2	24.5	29.3	28.0	27.6	27.2	23.9	22.0
Domain 3	31.4	37.7	35.1	35.3	37.6	32.0	29.4
Domain 4	37.3	45.2	41.9	41.6	46.5	38.4	35.8
Domain 5	42.6	52.1	47.7	47.9	53.0	44.3	41.7
Domain 6	47.6	58.0	53.1	53.7	58.6	49.5	47.1
Domain 7	52.3	63.4	58.0	59.0	64.0	54.3	52.1
Domain 8	56.7	68.3	62.6	63.9	69.1	58.8	56.9

^a^Continuous variables are scaled to unit variance; binary and categorical variables are transformed and then scaled using multiple correspondence analysis (MCA)

A similar picture emerged from the multitrait-multimethod analysis, with a number of variables showing relatively weak correlations with the domains to which they had been assigned based on the narrative review and modest ones with theoretically unrelated domains (see Table 4). For example, road quality had a correlation of just 0.13 with the domain of outdoor spaces and buildings, but a correlation of 0.35 with the domain of communication and information.

**Table 4:** Intra-domain^a^ and inter-domain correlations of community-level healthy-ageing indicators^b^

	**Domain**			
**Indicator**	**A**	**B**	**C**	**D**
***Domain A: Outdoor Spaces and Buildings***				
Sidewalk completeness	**0.31**	0.23	0.38	0.23
Presence of street trees & flowerbeds	**0.28**	0.11	-0.04	0.11
Access to parks & recreational areas	**0.10**	0.18	0.17	0.14
No. of physical-activity & recreational facilities	**0.11**	-0.02	0.04	-0.04
Road completeness	**0.13**	0.06	0.29	0.25
Road quality	**0.13**	0.20	0.35	0.25
Street lighting	**0.11**	0.14	0.22	0.15
Traffic lights	**0.21**	0.22	0.48	0.27
***Domain B: Transportation***				
Bus connections	0.18	**0.23**	-0.03	0.12
Train connections	0.10	**0.38**	0.06	0.22
Access to train stations	0.00	**0.42**	0.25	0.39
***Domain C: Communication and Information***				
Home internet	0.04	0.14	**0.53**	0.21
Free public internet	-0.12	0.11	**0.53**	0.01
***Domain D: Community Support and Health Services***				
Access to hospitals	0.08	0.29	0.01	**0.42**
Access to public medical clinics	0.06	0.17	0.15	**0.10**
Access to private medical clinics	0.11	0.27	0.19	**0.34**

^a^Intra-domain loadings are highlighted in bold

^b^Community social cohesion and civic participation and employment were excluded from these analyses as single-indicator domains

Taken together with the FAMD analysis, this pattern of results highlights the complexity of the relationships among indicators and indicates that developing domain-based scores or deriving an overall index via this approach is unlikely to appropriately describe community conditions related to healthy ageing within the PURE study sample. Although additional steps in the FAMD process could be used to statistically derive a set of study-specific domain scores, any such derived variables would fail to reflect the substantial variation by both community-level urbanness and country-level income. The integration of these variables in the planned epidemiologic analyses could subsequently result in differential exposure misclassification, introducing bias above and beyond purely random measurement error [30].

As a result, a decision was made to proceed with a qualitative assignment of individual indicators to six domains by achieving consensus among the three lead authors (EJR, CKC, and SAL) for the purpose of clarifying the relationship between indicators of age-friendly environments within PURE and the WHO age-friendly cities framework (see Table 4): 1) ***outdoor spaces and buildings:*** sidewalk completeness, presence of street trees and flowerbeds, access to public parks and recreational areas, number of public places for recreation or physical activity, road completeness, road quality, street lighting, and traffic lights; 2) ***transportation:*** connections to other towns via buses, connections to other towns via trains, and access to train stations; 3) ***social participation:*** community social cohesion; 4) ***civic participation and employment:*** access to government buildings; 5) ***communication and information:*** internet access at home and free public internet access; and 6) ***community support and health services:*** access to hospitals, access to public medical clinics, and access to private medical clinics.

Supplemental Fig. 1 depicts the alignment between the domains included in the original WHO guidelines, the other relevant environmental audit tools for healthy ageing, and this final set of PURE healthy-ageing indicators.

## Discussion

This study explored multiple approaches to developing robust indicators of age-friendly neighbourhood environments with potential application to urban and rural communities in countries with a wide range of national-income levels. Using existing measures of community environments collected within a global epidemiological study, we successfully adapted these measures to define and describe a novel set of indicators aligned with the World Health Organization’s age-friendly cities framework [12]. In the context of a longitudinal study for which recruitment began five years before the publication of the WHO framework, we were able to address six of the original eight WHO domains: 1) outdoor spaces and buildings; 2) transportation; 3) social participation; 4) civic participation and employment; 5) communication and information; and 6) community support and health services. However, we were unable to include indicators for two domains — 1) housing; and 2) respect and social inclusion — which are among those most commonly excluded from comparator audit tools as well, included solely in an attempt to apply the WHO criteria to informal settlements in Nairobi, Kenya [15].

The current effort builds upon the WHO’s pilot-testing process, which was carried out across locations representing a range of population densities, cultures, and demographic profiles in 2014–2015. That study reported an average of 24% fidelity between the standard indicators and available metrics across these sites, citing difficulties with data collection as the principal issue impeding the use of the indicators [31]. Although the specific indicators available for use in PURE vary from those recommended by the WHO, they align with 75% of the broad domains included in the original WHO age-friendly cities framework. The geographic scope of the current project is also considerably larger, reflecting 496 communities located in 20 countries, as compared to the 15 communities across 11 countries included in the WHO’s pilot tests [31].

In addition, our findings regarding the impact of urbanness complement existing efforts to extend the WHO framework from urban regions to rural and remote areas, such as that carried out via a series of focus groups held in ten communities across Canada in 2007 [16]. This project highlighted several features of greater importance to residents of rural and remote areas than urban ones, particularly driving safety, expanded public transportation, alternative channels for information provision, and the creation of a “one-stop shop” to provide healthcare and other support services in a single, accessible location [16]. Although this phase of our study was not designed to assess the importance of individual indicators or broader domains to specific health outcomes, it demonstrates striking differences in access to supports for healthy ageing by level of urbanness, including in specific areas such as public-transit availability and access to government services highlighted by the Canadian effort [16].

In this way, our effort builds upon prior studies that have cited the importance of urbanness to healthy ageing without examining its relationship to specific indicators. For example, while developing the Neighbourhood Design Characteristics Checklist (NeDeCC) in England, Burton et al. reported that the urban–rural status of older adults’ residences had one of the strongest associations with well-being of all 25 included indicators, but the researchers were unable to examine variation in the other indicators by urbanness due to sample-size limitations [21]. The creators of the Older People’s External Residential Assessment Tool (OPERAT) reported significantly higher scores in the domains of natural elements and incivilities and nuisance in the most-urban environments, but noted their small, non-random sample of 500 adults and their focus on Wales alone as important limitations [22]. An effort to adapt the WHO framework to both urban and rural communities across China by integrating data from the China Health and Retirement Longitudinal Study (CHARLS) was explicitly designed to examine variation by urbanness and found that all indicators of age-friendliness were more common in urban areas across the six included domains (see Supplemental Fig. 1), similar to our own outcome [14]. However, their extensive adaptation of individual indicators means these results are not directly comparable to ours. Looking beyond the healthy-ageing literature, our findings regarding urbanness and public-transit availability align with those reported in a study that applied an adapted version of the EPOCH 1 tool to assess community-level features associated with CVD risk factors in 2,074 urban and rural communities across Canada, which similarly found significantly lower availability of buses and trains in rural settings than in urban ones [32].

Research examining the relationship between country-level income and environmental indicators related to healthy ageing is rare. In fact, none of the comparator tools identified in our scoping review integrated data from more than one low- or middle-income country [14, 15, 23], except the efforts led by the WHO itself [12, 13]. However, neither of the two WHO efforts were designed to assess variation in the availability of indicators by country-level income, preventing any direct comparison. We identified substantial variations in the individual indicators in domains 1 and 2 by country-income class, suggesting that healthy-ageing indicators need to be adapted to specific resource levels and contextual settings. For example, using a single distance to train and bus stations to define public-transit availability in both high- and low-income countries overlooks the fact that residents of low-income countries are less likely to have access to a vehicle to travel to such a station [33], reducing the maximum distance that reflects practical accessibility.

 All in all, the results of both the FAMD and MTMM analyses and the availability of individual indicators across the diverse set of communities included in this study support calls in the existing literature to abandon uniformity in favour of complexity. In fact, the WHO’s guide to using the core indicators of the AFC framework states the guidelines are “﻿something to be adapted, as necessary and appropriate, to build an indicator set that is most meaningful and relevant in the local context” [13], and a number of the studies identified in our narrative review described such adaptation to lower-income countries [15] and rural communities [14, 16]. Parallel efforts have generated indicators using local data rather than the WHO AFC framework, including the Multidimensional Assessment System of the Built Environment (MASBE), which was refined using case studies in Mexico and Spain [34], and the ﻿Age-friendly Urban Index (AFUI) in Ireland, which used confirmatory factor analysis to identify three domains and calculate a single score [29]. In addition, numerous projects have narrowed in on specific aspects of age-friendliness, such as accessibility and protection from harmful exposures [35]. These include the ﻿Mobility Over Varied Environments Scale (MOVES) tool, developed using data from a population-based survey of older Canadians [36], and the Senior Walking Environmental Assessment Tool (SWEAT), which measures features related to physical activity [37]. However, because the WHO framework is so widely applied — the WHO Global Network for Age-friendly Cities and Communities comprised 1,114 sites home to more than 262 million individuals in late 2021 [38] — adapting and applying the full set of the WHO’s AFC indicators across the broadest possible range of settings remains critically important [39].

 Looking forward, this project provides the foundation for applying these indicators to multiple domains of healthy ageing among adults aged 50 and older within PURE’s unique study cohort. Critically, PURE’s longitudinal design will advance the exploration of complex causal pathways that link exposures recorded between 2010 and 2015 to outcome data captured in follow-up surveys completed through 2021. Three major epidemiologic studies are planned, each building on the prior effort. The first will examine social isolation based on a scale previously developed for PURE analyses and comprising marital status, social support, and group membership [40]; the second will look at three distinct measures of mental health (stress, depression, and suicide); and the third and final study will evaluate incident CVD and CVD mortality. In addition, future funding will be sought to repeat the EPOCH assessments using the same tools, expanded to include all of the domains recommended by the WHO. This process will allow us to document changes in community-level healthy-ageing indicators over time and then relate these shifts to changes in risk factors and measures of social, psychological, and physical functioning.

### Strengths and limitations

The timespan of EPOCH data collection represents one of this study’s major limitations: because we collected environmental data for each community over a relatively short timeframe, the results presented here may not reflect the current age-friendliness of PURE study communities. However, a new round of EPOCH data collection is planned to update each community’s rankings and to examine changes over time. Conversely, the fact exposure data pre-date outcome data is a key strength of the planned epidemiologic studies. The planned data-collection effort could also help overcome another limitation of the current study: the fact that the tools were not developed specifically with age-friendliness in mind. This precluded our ability to assess whether public spaces, buildings, or public-transit vehicles are accessible to older adults with mobility, vision, or hearing limitations, a factor that is highlighted throughout the WHO criteria [13].

The EPOCH data collection that informs the current analysis was also limited in its geographic scope, with the bulk of the variables based on a systematic social observation conducted throughout a one-kilometre walk. Because the precise latitude and longitude of the centre of each PURE community has been recorded, however, one potential method of overcoming these limitations is the integration of similar indicators from satellite or other georeferenced data, which have become significantly more widely available over the past decade for the areas under study.

In addition, although participant observations inform several indicators, not all study participants were older adults. Earlier efforts that have integrated subjective assessments among members of this age group have demonstrated the utility of such an approach, particularly to rank [16] or weight [22, 34] objective indicators or for the assessment of indicators for which objective data may be lacking, such as accessibility to buildings by wheelchair users [15]. However, a number of similar environmental audit tools failed to include any input from members of this age group [14, 21], making our approach an advancement over these others.

Perhaps the biggest limitation of this study is the fact that our indicator selection was confined to a single set of existing definitions applied universally across PURE’s diverse communities. Our analyses demonstrate substantial variation by both community-level urbanness and country-level income, indicating that the ideal construction of healthy-ageing indicator variables should take these moderating factors into account. For example, although EPOCH 1 defines access to a range of resources (such as government buildings and train stations) via a 20-km distance from the community centre, this single linear distance may equate to widely varying travel times in urban vs. rural locations; older adults’ sense of perceived accessibility is also likely to differ based on geographic and cultural factors [11].

Finally, although we were able to address the bulk of the broad domains identified by the WHO’s healthy-ageing indicators framework, the precise indicator definitions differed significantly. This last aspect of our study design will limit our ability to speak specifically to the relationship between the WHO’s age-friendly criteria and the health outcomes captured in PURE, but this concern is offset by the broad geographic scope, large sample size, and diversity of the PURE cohort.

## Conclusions

Our narrative review indicates that very few earlier efforts have examined variation in access to supports for healthy ageing across urban and rural communities. Further, little is known among countries with varying national-income levels and across a diverse set of geographic regions, making it difficult to gauge the extent to which the supports outlined in the WHO age-friendly cities framework are relevant to specific communities or how best to adapt them to more fully reflect the local context. Enhancing the ability to connect distinct community features to multiple aspects of healthy ageing will support the development of interventions tailored to the public-health priorities of individual communities. In addition, identifying how these relationships may vary in areas with more significant disadvantages or racialized communities will provide a foundation for promulgating age-friendly policies and designs that maximize overall population-health benefits without exacerbating well-known health inequities, a necessary step to achieve distributional justice [41]. Finally, clarifying these connections will support the integration of the age-friendly cities construct along with other policy paradigms such as healthy cities [42] and the health in all policies (HiAP) approach [43], which do not explicitly account for differential impacts on or preferential designs for older adults, helping to extend any potential health benefits to individuals of all ages.

## Supplementary Information

**Additional file 1: Fig. 1. **Domain comparison across environmental audit tools for healthy ageing.

**Additional file 2: Table 1. **Description of EPOCH 1 and EPOCH 2 variables with relevance to age-friendly neighbourhood environments.

## Data Availability

The datasets analysed during this study are not publicly available because the various centre-specific ethics committees governing the underlying data sources have not given permission to share them publicly and the study is ongoing. These data are available from the corresponding author upon reasonable request.
