# Supplementary material for: Developing indicators of age-friendly neighbourhood environments for urban and rural communities across 20 low-, middle-, and high-income countries
Source: BMC Public Health. 2022 Jan 13;22:87. doi: 10.1186/s12889-021-12438-5 (PMC8759164; doi:10.1186/s12889-021-12438-5)
Supplement: Supplementary file 1 — Additional file 1: Fig. 1. Domain comparison across environmental audit tools for healthy ageing. [file 12889_2021_12438_MOESM1_ESM.docx]

**Supplemental Figure 1.** Domain comparison across environmental audit tools for healthy ageing

|  | **WHO, 2007** | **WHO, 2015** | **Burton et al, 2011** | **Burholt et al, 2016** | **Aboderin et al, 2017** | **Wang et al, 2017** | **Ralston, 2018** | **PURE Healthy Ageing** |
| --- | --- | --- | --- | --- | --- | --- | --- | --- |
| **Outdoor Spaces & Buildings** | **🗸** | **🗸** | **🗸** | **🗸** | **🗸** | **🗸** | **🗴** | **🗸** |
| **Transportation** | **🗸** | **🗸** | **🗸** | **🗸** | **🗸** | **🗸** | **🗸** | **🗸** |
| **Housing** | **🗸** | **🗸** | **🗴** | **🗴** | **🗸** | **🗸** | **🗴** | **🗴** |
| **Social Participation** | **🗸** | **🗸** | **🗴** | **🗴** | **🗸** | **🗸** | **🗴** | **🗸** |
| **Respect & Social Inclusion** | **🗸** | **🗸** | **🗴** | **🗴** | **🗸** | **🗴** | **🗴** | **🗴** |
| **Civic Participation & Employment** | **🗸** | **🗸** | **🗴** | **🗴** | **🗸** | **🗴** | **🗴** | **🗸** |
| **Communication & Information** | **🗸** | **🗸** | **🗴** | **🗴** | **🗴** | **🗸** | **🗴** | **🗸** |
| **Community Support & Health Services** | **🗸** | **🗸** | **🗴** | **🗴** | **🗸** | **🗸** | **🗸** | **🗸** |
