# Supplementary material for: Developing indicators of age-friendly neighbourhood environments for urban and rural communities across 20 low-, middle-, and high-income countries
Source: BMC Public Health. 2022 Jan 13;22:87. doi: 10.1186/s12889-021-12438-5 (PMC8759164; doi:10.1186/s12889-021-12438-5)
Supplement: Supplementary file 2 — Additional file 2: Table 1. Description of EPOCH 1 and EPOCH 2 variables with relevance to age-friendly neighbourhood environments. [file 12889_2021_12438_MOESM2_ESM.docx]

**Supplemental Table 1.** Description of EPOCH 1 and EPOCH 2 variables with relevance to age-friendly neighbourhood environments

| **Tool** | **Variable Type** | **Variable Description** |
| --- | --- | --- |
| EPOCH 1 | Binary | Access to a public park or recreation area within 20 km of centre* |
| EPOCH 1 | Binary | Access to a secondary school within 20 km of centre |
| EPOCH 1 | Binary | Access to a university within 20 km of centre |
| EPOCH 1 | Binary | Access to a post office within 20 km of centre |
| EPOCH 1 | Binary | Access to a bank within 20 km of centre |
| EPOCH 1 | Binary | Access to a government building within 20 km of centre* |
| EPOCH 1 | Binary | Access to a train station within 20 km of centre* |
| EPOCH 1 | Binary | Access to a police station within 20 km of centre |
| EPOCH 1 | Categorical | Sidewalk completeness  (none, partial, complete on one side, complete on both sides)* |
| EPOCH 1 | Categorical | Sidewalk quality (poorly maintained, undergoing repair, somewhat maintained, well maintained)* |
| EPOCH 1 | Binary | Presence of electrical street lighting* |
| EPOCH 1 | Binary | Presence of traffic lights* |
| EPOCH 1 | Categorical | Road completeness (no paved roads, partially paved, completely paved)* |
| EPOCH 1 | Categorical | Road quality (poorly maintained, undergoing repair, somewhat maintained, well maintained)* |
| EPOCH 1 | Numeric | Number of places for recreation or physical activity* |
| EPOCH 1 | Numeric | Number of street trees and flowerbeds* |
| EPOCH 1 | Numeric | Distance to nearest restaurant from centre |
| EPOCH 1 | Numeric | Distance to nearest pharmacy from centre |
| EPOCH 1 | Numeric | Number of public buses available per day* |
| EPOCH 1 | Numeric | Number of public trains available per day* |
| EPOCH 1 | Binary | Connected to other towns/cities by bus via regular service* |
| EPOCH 1 | Binary | Connected to other towns/cities by train via regular service* |
| EPOCH 1 | Numeric | Cost per unit area of residential land* |
| EPOCH 1 | Numeric | Average house cost* |
| EPOCH 1 | Binary | Access to a hospital within 20 km of centre* |
| EPOCH 1 | Binary | Access to a public medical clinic within 20 km of centre* |
| EPOCH 1 | Binary | Access to a private medical clinic within 20 km of centre* |
| EPOCH 2 | Numeric | How many minutes does it take to get to your grocery? |
| EPOCH 2 | Categorical | How do you usually get to the grocery? (walk, bicycle, car, bus, train, motorcycle, other personal motorized vehicle, other public transport) |
| EPOCH 2 | Categorical | How do you usually get to work? (walk, bicycle, car, bus, train, motorcycle, other personal motorized vehicle, other public transport) |
| EPOCH 2 | Categorical | In your opinion, do people generally help others not related to them in this community? (this is common for people in our neighbourhood; some adults would do this in our neighbourhood; infrequently, but it may happen; this would not occur in our neighbourhood)* |
| EPOCH 2 | Binary | Do you have access to the internet at home?* |
| EPOCH 2 | Binary | Do you have access to the internet in the community for free?* |

*Indicator included on initial list determined through consensus
